# Job preferences of medical and nursing students seeking employment in rural China: a discrete choice experiment

**DOI:** 10.1186/s12909-021-02573-3

**Published:** 2021-03-05

**Authors:** Meiling Bao, Cunrui Huang

**Affiliations:** 1 School of Public Health, Sun Yat-sen University, Zhongshan Road #2, Guangzhou, 510080 China; 2 School of Public Health, Guizhou Medical University, Guiyang, China; 3 School of Public Health, Zhengzhou University, Zhengzhou, China

**Keywords:** Discrete choice experiment, Job preferences, Health workers, Medical and nursing students, Recruitment policy

## Abstract

**Background:**

China has a shortage of health workers in rural areas, but little research exists on policies that attract qualified medical and nursing students to rural locations. We conducted a discrete choice experiment to determine how specific incentives would be valued by final–year students in a medical university in Guizhou Province, China.

**Methods:**

Attributes of potential jobs were developed through the literature review, semi–structured interviews, and a pilot survey. Forty choice sets were developed using a fractional factorial design. A mixed logit model was used to estimate the relative strength of the attributes. Willingness to pay and uptake rates for a defined job were also calculated based on the mixed logit estimates.

**Results:**

The final sample comprised 787 medical and nursing students. The statistically significant results indicated “Bianzhi” (the number of personnel allocated to each employer by the government) and physical conflicts between doctors and patients were two of the most important non-monetary job characteristics that incentivized both medical and nursing students. Policy simulation suggested that respondents were most sensitive to a salary increase, and the effect of incentive packages was stronger for students with a rural family background.

**Conclusions:**

Strategies for patient–doctor relationships, Bianzhi and salary should be considered to attract final–year medical and nursing students to work in rural China. In addition, specific recruitment policy designs tailored for students with different majors and backgrounds should be taken into account.

**Supplementary Information:**

The online version contains supplementary material available at 10.1186/s12909-021-02573-3.

## Introduction

The uneven distribution of health workers reduces access to essential health services and contributes to inequalities in health outcomes [1]. Studies have cast light on the systematic categories of imbalances in the health workforce that affect the medical system, including geographic, institutional, professional and ownership imbalances [2]. Of these imbalances, the paucity of health workers in disadvantaged areas has the most significant bearing on universal health coverage and adversely affects the health of individuals living in such areas [3]. Disparity between urban and rural areas in terms of health workforce has become a critical health policy concern in many countries [4, 5].

China’s higher education system reform was launched in 1998, which has led to a major expansion of universities and has facilitated fast growth in the educational sector [5]. However, faculty numbers in medical institutions have not kept pace with the expansion in the number of students enrolled at these institutions [6]. Although China’s total health workforce rose remarkably, from 6.7 million people in 2006 to 10.2 million in 2014 [7], it failed to meet the demand by 500,000 physicians in rural areas [8]. In addition, the low nurse to doctor ratio (1.1,1) constrains the development of clinical practice to some extent [7]. Further, the density of nurses in urban areas is much higher than in rural areas, and the gap is still widening [9].

The job preferences of undergraduate medical and nursing students will influence the geographical distribution of health workers in the future. Accordingly, it will be necessary to elicit specific policy incentives targeted at final–year medical and nursing students to raise the attractiveness of posts in rural areas usually considered less desirable due to the heavy workload, poor infrastructure and inconvenient transportation [10]. The World Health Organization (WHO) has identified initiatives such as educational interventions, regulatory interventions, financial incentives, and personal and professional support packages to attract health workers to take up positions in disadvantaged areas [11]. WHO has also proposed that countries identify appropriate interventions suitable to their local contexts, given the diversity in local demand factors as well as of health professionals and the specific characteristics of each labor market [12]. Thus, the development of such strategies requires precise insight into the job preferences of health professionals in various countries.

Implications for policy can be derived from the use of a discrete choice experiment (DCE), which has been used in health economics research to determine the job preferences of health workers. A user guide on how to conduct a DCE for health workforce recruitment and retention in rural areas was developed by WHO and two other agencies [13]. The choice experiments are conducted by using theories of demand and random utility [14], assuming that the utility associated with a good or service is made up of the utilities of its attributes [15]. It is a well–established approach for identifying the relative value that people place on factors (attributes) [16]. Although individuals want the best of all attributes associated with a job, resources constraints may prohibit such a choice. In such a case, the DCE provides a weighed relevance to the attributes to distinguish highly valued ones. Therefore, quantitative information on the relative strength of selected attributes can be determined, and trade–offs between these attributes and the probability of individuals taking up these jobs can be ascertained [13, 17].

In China, health professionals have had to confront the challenges associated with the economic and medical reforms. For example, difficulties in human resource management like the “Bianzhi” system continue to exist. China had a special planned employment system from 1949 to 1978 called “Bianzhi”, in which the government decided the number of personnel allocated to each institution. After the market economic reforms initiated in 1980s, this system gave way to contract–based employment system. However, even after the introduction of contract–based work, Bianzhi positions are highly valued by the Chinese people because of additional benefits of the position and the sense of belonging it fosters [18]. Moreover, a new problem that has emerged in China is violence against medical staff which seriously affects how doctors and patients interact, and led to a widespread concern [19]. In our study, both patient–doctor relationships and Bianzhi were included as factors to identify their impact on the job preferences of medical and nursing students.

Although a few DCE studies in China that explored health workers’ decisions about accepting rural jobs found that the income level, benefits of work, training opportunities, children’s education, and the level of respect received from the community were some important influencing factors [20, 21], there is a paucity of evidence about the role of incentives in undergraduates’ rural work decisions. To our knowledge, this is the first study to explore the impact of patient–doctor relationships on rural recruitment. We aimed to explore factors likely to affect the job preferences of final–year medical and nursing students in order to assist policy makers in designing interventions to attract qualified students to rural areas.

## Methods

### Study setting and sampling

This research was conducted in Guizhou Province for three reasons. First, Guizhou is one of the most underdeveloped provinces in China, with the penultimate rank for urbanization. Second, rural residents made up 56% of the population, whereas only 30% of total health workers were posted in rural areas. Third, the density of the province’s licensed doctors was 1.94 per 1000 residents compared to 2.31per 1000 residents in the country as a whole. Similarly, the density of nurses was 2.42 per 1000 residents, compared to the national average level of 2.54 per 1000 residents [22].

In semi–structured interviews we conducted in August 2017, health officials in Guizhou Province identified doctors and nurses as the medical personnel in most demand to run primary health facilities effectively. Guizhou Medical University was selected because it was the top-ranked medical college and had the most graduates in the province. The target group of our research was final–year medical students in five–year programs and nursing students in three–year and four–year programs who had undergone clinical training. It is assumed that they are considering career options but have not made their decisions. Anywhere between 20 and 50 respondents per experiment group are required to reliably estimate respondent preferences [23]; thus, all final–year medical and nursing students were invited to participate in the study (excluding the 33 students who participated in the pilot test).

### Identification and selection of attributes and levels (instrument development)

DCE is an evaluation method founded on the random utility theory. This theory requires a multiple attribute approach that consists of breaking down the examined good into its different components or attributes for which the levels vary systematically between choice sets [24, 25]. The first stage of developing a DCE tool involves identifying attributes and then determining the levels of these attributes. Fifteen likely attributes were identified through a literature review, which were then narrowed down through semi–structured interviews conducted with 23 doctors and 18 nurses who were present on the day of the visit from nine primary health facilities in Guizhou province. To ensure common views were captured and geographical representation, we conducted the interviews in four counties and one district during July in 2017(Table S1). Semi–structured interviews were conducted with six senior government officers in the Health Commission of Guizhou Province in charge of the primary healthcare to identify the process through which policy incentives are realistically developed. Once the interviews were digitally recorded and transcribed, we used thematic analysis and constant comparison to analyze the data. We also conducted a content analysis of existing policy documents. Prior to the data collection phase, DCE instruments were pretested with final–year medical students and minor modifications were made.

Eight attributes warranted inclusion in the final DCE (see Table 1): salary, educational opportunities, transportation, job location, workload, essential equipment, patient–doctor relationships, and Bianzhi. Salary was the monthly net salary after taxes. A salary attribute was always included in the DCE to estimate of the willingness to pay (WTP) for improvements in other attributes [21]. Given the hypothetical nature of the levels of attribute, and in order to compare the job preferences between medical students and nursing students, the difference in practical salary between the two groups was not shown in this study. The lowest salary level included in the instrument represented the average salary estimated through the interviews with health officials.

Educational opportunities represented only the training opportunities medical professionals could use to improve their clinical skills. The transportation attribute was considered “inconvenient” if the round trip between workplace and residence took more than 1.5 h, and “convenient” if it took 30 min or less. Job location was determined according to the administrative divisions established by the government. Villages and towns corresponded to rural areas, whereas cities corresponded to urban areas. We also assumed that working 50 h per week was common for rural health workers based on interviews with health workers and officers mentioned above, so was the reference level. Essential equipment was regular medical equipment/facilities suitable for common and frequently–occurring conditions.

Because there were reports of escalating tensions between doctors and patients in China [19], and health officials in Guizhou Provincial Health Commission suggested that we include this attribute in the DCE, patient–doctor relationships was included. As one level of this attribute, suit (synonymous with the terms “lawsuit” or “litigation” as relevant throughout the document) meant that the medical dispute needed to be settled in court.

Positions that provide life–time job security, benefits and a sense of belonging are called Bianzhi or “iron rice bowl” positions, whereas other positions are based on short term contracts [18]. In order to be clear and not confuse the respondents, most of the attributes were measured quantitatively. For example, we used frequencies (e.g., once a year) instead of qualitative measurements (e.g., insufficient, some, sufficient) to measure educational opportunities.

### Experimental design and choice set construction

As described in Table 1, there were two attributes with four levels, two attributes with three levels and four attributes with two levels. This design generated 2304 (4^2^ × 3^2^ × 2^4^) potential scenarios with different combinations of levels of the eight job attributes and 2,653,056 ((2304 × 2303)/2) potential choice tasks. The number of potential scenarios presented to the respondents was reduced by a fractional factorial experiment design, and the %MktRuns macros of SAS 9.4 was used to optimize the D-efficiency, minimize the overlap among attributes levels, and maximize level balance and orthogonality. Furthermore, a restrictions macro was also used to avoid dominated alternatives [26], dominance in a DCE means a participant always selected job scenarios on the basis of one attribute.

**Table 1:** Posting attributes and levels used in discrete choice experiment

Attributes	Levels
Education opportunity	Once every 5 years
	Once every 5 years
	Once a year
Transportation	Inconvenient
	Convenient
Salary^a^	RMB¥3000/month
	RMB¥5000/month
	RMB¥7000/month
	RMB¥9000/month
Job location	Villages and towns
	City
Workload	50 h per week
	40 h per week
	60 h per week
Essential equipment	Inadequate
	Adequate
Patient–doctor relationships	No (quarrel, physical conflict and lawsuit)
	Quarrel
	Physical conflict
	Lawsuit
Bianzhi	No Bianzhi
	Have Bianzhi

^a^For analysis, salary was treated as a continuous variable. All other attributes were dummy coded. A base level was defined to reflect the current labor market conditions, whereas other levels were modifications from that base level

The final design consisted of 40 sets. A systematic review identified that previous DCEs administered to health workers used 16–20 choice tasks [27]. In order not to exhaust the respondents, the 40 sets were blocked into two questionnaire versions, each containing 20 choice sets. The blocks were randomly allocated to the respondents. Each choice set comprised two job scenarios (Job A and Job B; see Figure S1). A forced choice approach was employed to elicit more information about respondents’ preferences for attributes; since students had no empirical understanding of the job in reality, the opt–out option (to choose their current job) was not included.

We used an unlabeled DCE because some evidence suggested that labels may distract respondents from job attributes and thus, diminish the reliability of the job preferences estimates [28]. The questionnaire was presented in three sections: an introductory script to acclimate respondents to the hypothetical nature of the DCE they were about to take (including the telephone number of a research group member if interviewees had problems with questionnaires); a set of socio–demographic characteristics questions; and 20 choice sets. The survey was conducted between October and December 2017.

### Survey administration

The self–administered questionnaire was emailed to the class tutor who uploaded it to Class QQ Population (instant messaging software, widely used in China). The questionnaire was then downloaded by students who sent it to the class monitor after finishing it. The class monitor collected, packed, and emailed the questionnaires to the research group.

### Pilot-testing

Prior to the start of data collection, we conducted a pilot study with 33 medical students. This process provided an opportunity to determine whether the presentation of the questionnaire was conceptually clear. Minor corrections were made to the questionnaire to enhance the readability, and respondents reacted positively to the survey, completing it within 20 min.

### Statistical analysis

All data collected from the DCE questionnaires was entered and stored using Microsoft Excel 2010 (Microsoft Corporation, USA). Descriptive statistics were calculated for demographic variables, and the mixed logit models were adopted to analyze the DCE data. Mixed logit models take the preference heterogeneity among respondents into consideration, making it a popular choice to conduct research [1, 9, 14, 25, 29–37].

All attributes variables were coded as dummy variables except for salary, which was specified as a continuous variable to facilitate the calculation of WTP. WTP estimates were calculated by dividing attribute coefficients by the salary coefficient for each model, conveying in monetary terms respondents’ preferences for one level of an attribute as compared to the reference level. Analysis on the valuation and comparison of different policies was offered by calculating uptake rate of a defined job [38]. Sex and rural–urban background information was then used to divide the sample into subgroups and a separate analysis on each subgroup was then carried out. All mixed logit models were fit using StataMP 14’s mixlogit command (Stata Corporation, USA), and were specified with500 Halton draws.

Several validity tests were conducted to determine the appropriateness of model specifications. The theoretical validity of the model was assessed by determining whether the coefficients were of the anticipated sign. We repeated the analysis using conditional logit models (all data available from the authors). In this study, the results from each conditional logit model were not substantively different from the mixed logit model. As for the external validity test, students volunteered their phone numbers at the end of the questionnaire, so we could contact them in the future and ascertain the actual choices they made after graduation.

### Ethics approval and consent to participate

This study was approved by the Medical Ethics Committee of Guizhou Medical University and undertaken with permission from the Health Information Center of Guizhou Provincial Health Commission. And we confirm that all methods were performed in accordance with the relevant guidelines and regulations. Participants were informed about the research, and gave informed consent prior to study participation. Respondents participated on a voluntary basis.

## Results

Of the 1168eligible final–year medical and nursing students at Guizhou Medical University, 879 (75%) agreed to participate in the survey. Four hundred thirty-eight were medical students and 441were nursing students. The response rates were 61 and 97%, respectively. Incomplete questionnaires were excluded from the sample. The final sample comprised 787 respondents, including 388 medical students and 399 nursing students.

The mean age of students was 24 years old (SD = 1.3), with ages ranging from 21 to 29 years. Nursing students were predominantly female (92%), while 58% of students majoring in clinical medicine were female. Meanwhile, 52% of medical students were from a rural background, and 66% of nursing students had a rural background (Table 2).

**Table 2:** Characteristics of participants (*N* = 787)

Variables	Medical students		Nursing students	
	frequency	%	frequency	%
*N*	388		399	
Sex				
Male	163	42	30	8
Female	225	58	369	92
Age group				
21–25 years old	341	88	368	92
26–29 years old	47	12	31	8
Background				
Urban	188	48	134	34
Rural	200	52	265	66

Outputs from two mixed logit models are represented in Table 3. The signs on all estimates were as expected, which implies that respondents derived a higher level of utility from the superior attribute level and made rational choices [15]. A workload of 60 h per week and unharmonious patient–doctor relationships had an adverse effect on preferences, while all the other attributes had a positive effect. Attributes were statistically significant at the 5% level (with the exception of “once every two years” for educational opportunities), indicating that they did have an impact on the probability of choosing a job.

**Table 3:** Mixed logit model results for medical and nursing students’ job preferences

Attributes	Parameter	Medical students		Nursing students	
		Coefficients ^**a**^	SE	Coefficients	SE
Salary ^b^					
Salary	Mean	0.000285***	0.00	0.000290***	0.00
Education opportunity ^c^					
Once every 2 years	Mean	0.035	0.043	0.061	0.039
	SD	0.002	0.091	0.120	0.163
Once a year	Mean	0.161***	0.046	0.107**	0.042
	SD	0.139	0.115	0.060	0.110
Transportation ^d^					
Convenient	Mean	0.235***	0.037	0.359***	0.041
	SD	0.308***	0.055	0.475***	0.045
Job location ^e^					
City	Mean	0.218***	0.055	0.231***	0.049
	SD	0.371***	0.089	0.111	0.190
Workload ^f^					
40 h per week	Mean	0.153***	0.046	0.227***	0.044
	SD	0.332***	0.069	0.224***	0.085
60 h per week	Mean	−0.106**	0.043	−0.150***	0.038
	SD	0.013	0.105	0.054	0.138
Essential equipment ^g^					
Adequate	Mean	0.271***	0.040	0.277***	0.033
	SD	0.475***	0.045	0.284***	0.050
Patient–doctor relationships ^h^					
Quarrel	Mean	−0.351***	0.049	−0.255***	0.048
	SD	0.081	0.195	0.122	0.136
Physical conflict	Mean	−0.893***	0.064	−0.498***	0.056
	SD	0.714***	0.068	0.445***	0.071
Suit	Mean	−0.884***	0.071	−0.497***	0.054
	SD	0.830***	0.079	0.346***	0.089
Bianzhi ^i^					
Have Bianzhi	Mean	0.411***	0.046	0.454***	0.039
	SD	0.635***	0.047	0.465***	0.044
Constant		0.118	0.034	−0.059	0.034
**Number of respondents**		**388**		**399**	
**Number of observations**		**15,520**		**15,960**	
**Log likelihood**		**− 4464.6944**		**− 4583.6866**	
**LR chi-square**		**275.86**		**124.41**	
**Prob > chi-square**		**0.0000**		**0.0000**	

^***^P < 0.01, ^**^P < 0.05

*SD* Standard deviation, *SE* Standard error

^a^The coefficient represents the mean relative utility of each attribute conditional on other attributes in a choice set, while the standard deviation of the random coefficients reflects the degree of heterogeneity in respondent preferences for a given attribute

^b^Continuous variable; the coefficient represents the magnitude of increase in utility by having one extra RMB

^c^Compared with once every 5 years

^d^Compared with inconvenient transportation

^e^Compared with rural job location

^f^Compared with 50 h per week

^g^Compared with inadequate essential equipment

^h^Compared with no quarrel, physical conflict and suit

^i^Compared with no Bianzhi

Physical conflict, Bianzhi and the availability of essential equipment were major predictors of medical students’ preferences (*β* = − 0.893, *β* = 0.411, *β* = 0.271, respectively), while physical conflict, Bianzhi, and the convenience of transportation were major predictors of nursing students’ preferences (*β* = − 0.498, *β* = 0.454, *β* = 0.359, respectively). From the standard deviation of the regression coefficients, we found significant preference heterogeneity exits over the availability of transportation, workload of 40 h per week, the availability of essential equipment, physical conflict between patients and doctors, lawsuits and Bianzhi for both medical and nursing students. In addition, job location was an important attribute for both medical and nursing students, for which medical students exhibited heterogeneity in their preferences, whereas nursing students did not.

Table 4 shows the results of WTP calculation. Mean utility coefficients are the basis for the WTP estimations which can be compared across different groups [29]. These measures predict how much salary a final–year student is willing to sacrifice in exchange for an improvement in a particular job attribute. The WTP for patient–doctor relationships gave us a clear indication about the importance of a harmonious relationship between the two. Medical students had to be compensated with RMB¥3138 (USD$464) per month to take up a job with potential physical conflict, while nursing students expected a remuneration of RMB¥1719 (USD$254). Medical students and nursing students were willing to pay RMB¥1443 (USD$213) and RMB¥1566 (USD$231), respectively, in order to obtain Bianzhi. Meanwhile, students were willing to forego RMB¥954 (USD$141) in exchange for jobs with adequate essential equipment.

**Table 4:** Willingness to pay^a^ for medical and nursing students

Variable	Medical students	Nursing students
Education opportunity		
Once every 2 years	121 (177, 419)^b^	211 (−51, 474)
Once a year	566 (248, 885)	370 (86, 654)
Transportation		
Convenient	827 (572, 1081)	1237 (965,1510)
Job location		
City	766 (390, 1142)	797 (465, 1128)
Workload		
40 h per week	536 (216, 856)	783 (488, 1078)
60 h per week	−372 (− 667,-76)	− 517 (− 776, − 258)
Essential equipment		
Adequate	953 (674, 1232)	954 (725,1183)
Patient–doctor relationships		
Quarrel	− 1233 (− 1577, − 888)	− 880 (− 1199, − 561)
Physical conflict	−3138 (− 3588, − 2688)	−1719 (− 2097, − 1341)
Suit	− 3103 (− 3598, − 2609)	− 1716 (− 2085, − 1346)
Bianzhi		
Have Bianzhi	1443 (1123, 1763)	1566 (1302, 1830)

^a^RMB per month. RMB¥1 = USD$ 0.1479, in 2017

^b^95% confidence intervals in parentheses, the confidence intervals are calculated with the nlcom–command in Stata

An analysis of the medical students revealed that women expected to be compensated with more money than men to work in areas where the patient–doctor relationships were more likely to be tense. And with the exception of the availability of adequate essential equipment, men were more willing than women to sacrifice more of their salary in exchange for other attributes. Medical students from a rural background were willing to sacrifice more pay than those from an urban background in exchange for all attributes, except when it required them to move to an urban center (Table S2).

**Fig. 1:**
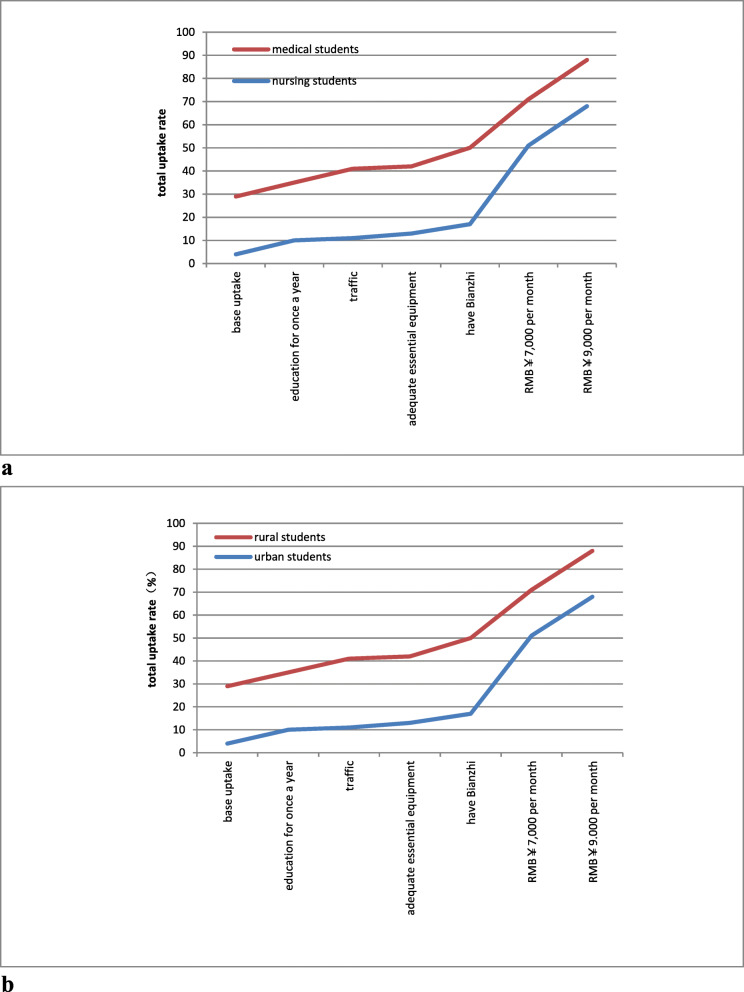
Changes of total uptake rate of taking a rural job under different intervention. **a** Medical students versus nursing students. **b** Rural background students versus urban background students

Figure 1 shows the likelihood of medical and nursing students as well as students from rural and urban backgrounds taking up rural jobs to measure the effectiveness of the different policies proposed to increase the attractiveness of those jobs. Not surprisingly, urban jobs were more strongly preferred than rural ones, especially among nursing students and those from urban backgrounds (the total uptake rate for rural postings at the baseline was 2 and 4%, respectively). It appears that an increased salary was the most effective incentive that could be offered to improve the attractiveness of rural posts among individuals of all four groups. As the salary level went up, the theoretical impact on rural recruitment would increase. As the simulation result suggests, an increase in the salary from RMB¥5000 to RMB¥7000, was associated with a corresponding increase in the probability of accepting the job posting by 19% for medical students. For intervention package with a salary of RMB ¥5000 (USD$739) per month, educational opportunities for once a year and having Bianzhi would be equivalent to a salary of RMB¥7000 (USD$1035) per month for medical students.

It is worth noting that incentive packages had stronger effects on students from rural backgrounds than those from urban backgrounds. Similarly, nursing students were more responsive to intervention policies, although they were less likely to choose rural jobs at the baseline. Table 5 shows the other intervention packages.

**Table 5:** Predicted impact of different policy interventions on uptake of rural postings

Intervention	Medical students		Nursing students		Rural background		Urban background	
	Change (%points)	Total uptake (%)	Change (%points)	Total uptake (%)	Change (%points)	Total uptake (%)	Change (%points)	Total uptake (%)
Base uptake		28		2		29		4
**Single interventions**								
Education for once a year	7	35	5	7	6	35	6	10
Convenient transportation	7	35	15	17	12	41	7	11
Salary RMB¥5000 per month	22	50	25	27	22	51	25	29
Salary RMB¥7000 per month	41	69	47	49	42	71	47	51
Salary RMB¥9000 per month	58	86	65	67	59	88	64	68
Workload of 40 h per week	6	34	9	11	6	35	7	11
Adequate essential equipment	12	40	12	14	13	42	9	13
Have bianzhi	15	43	20	22	21	50	13	17
**Intervention packages**								
Education+5000salary + Bianzhi	41	69	47	49	54	83	42	46
Transportation+5000salary + Bianzhi	42	70	54	56	61	90	43	47
Workload +equipment +Bianzhi	32	60	39	41	46	75	28	32
7000salary + equipment +Bianzhi	61	89	68	70	66	95	62	66
7000salary + equipment	51	79	56	58	60	89	54	58

Compared with a baseline job posting defined as: education for once every 5 years; inconvenient transportation; salary for RMB¥3000 per month; workload for 50 h per week; inadequate essential equipment; medical order for no quarrel, physical conflict and suit; no bianzhi

## Discussion

In the absence of well–designed studies using revealed preference data, we conducted a DCE among final–year health students to understand their preferences in job attributes. Effective interventions need to be matched with job preferences and expectations. In addition, it is vitally important to ensure that incentive policies aimed at healthcare workers are developed and evaluated on the basis of comprehensive empirical evidence. Our study provides a rigorous methodology through which empirical evidence can be evaluated. Further, DCEs are useful in that they can examine the likely effects of policies that have not been executed, thereby providing important information to guide the design of such incentives.

This DCE analysis offers important insights into the nature of the labor market for healthcare workers in China. We found that the job preferences of final–year healthcare students were affected by sufficient opportunities for education, the availability of adequate equipment, job location, lighter workloads, convenient transportation, harmonious patient–doctor relationships, Bianzhi posts, and job remuneration. This finding suggests that there could be a range of policy interventions to improve the probability of choosing a rural job.

Our findings are in agreement with the results of other studies in which educational opportunities, availability of equipment, workload, and convenient transportation were valued [14, 35, 39]. Educational opportunities offered once over a period of 2 years had no significant effect on the job selection process, whereas the opportunities offered annually had a significant effect, implying that policy should focus on the frequency of educational opportunities offered to attract students to rural areas. This finding echoes the DCE conducted with students in Tanzania, which revealed that they were eager to gain more knowledge [38]. Thus, the government should consider a program where healthcare workers in rural areas are guaranteed access to educational training courses at least once a year.

Additionally, we found that adequate health facility equipment is an important determinant of job choice. This is particularly true for medical and nursing students, who are often disappointed by the gap between the skillset they have acquired using the tertiary hospital infrastructure and the availability of equipment in rural health facilities. These results are consistent with those from other DCEs [12, 34, 36, 37, 40]. Moreover, a review paper on health workers’ motivation and retention indicates that the improvement of hospital infrastructure and resource availability could increase retention [41].

We also found that respondents wished to have adequate leisure time and there was some aversion to higher workloads. The results are in line with other DCEs [42–44]. It was likely an expression of a generational shift in attitudes. Our respondents had grown up after the reforms instituted in the 70s and 80s had taken effect, and they were unlikely to feel the sense of material deprivation experienced by previous generations. Students this age may not believe that they have to work as hard as previous generations for their subsistence, and they may be inclined to achieve a work–life balance with an adequate amount of free time. As a result, it will be necessary for the relevant authorities to account for the demands of a changing workforce by incorporating adequate time for rest, so as to make jobs in rural areas sufficiently attractive.

Both medical and nursing students placed a high value on patient–doctor relationships, a concern expressed by many parties. Patients have complained about the lack of communication and empathy shown by doctors, and, there have been reports of widespread physical attacks on doctors in China [45]. In this context, one study found that healthcare workers regarded the respect of the community as important [21]. The DCE information we present here may be used to inform the development of specific policy interventions. Given the high patient–doctor tension in China and the results of our studies, the introduction of guidelines for a humanistic practice and the inculcation of professional norms in medical schools is urgent and essential [7]. “Physical conflict” was the most disliked outcome for our respondents, followed by “lawsuit”, suggesting that these students place more value on their safety than on avoiding legal disagreements. Thus, in order to attract final–year students to take up positions in disadvantaged areas, regulations and institutions need to be improved to ensure their safety.

Not surprisingly, Bianzhi had a large effect on job preferences. This key finding has important policy implications. The majority of healthcare workers in China work at state–owned health facilities. The Bianzhi system provides workers with government identification and the corresponding benefits. Nursing students were willing to pay more money than medical students to obtain Bianzhi, partly because of the prevalence of contract–based nurses as opposed to Bianzhi nurses, and the inequities between them in terms of wages and benefits. In this regard, studies have found that disparities between contract labor and the permanent positions may have an adverse impact on the satisfaction levels of nurses and patients in hospitals [18]. Although such environments are hard to change, this substantial impact on job preferences should be considered in health policy discussions. For example, the government could emphasize “equal pay for equal work” as a strategy to eliminate the disparities between contract–based jobs and those sanctioned by the government.

Our findings confirm that financial incentives are very important in attracting final–year students to rural settings. This reinforces previous findings that health workers were not entirely satisfied with their salaries [32]. As medical students try to fulfill their fundamental needs, they are concerned about their livelihood [21]. From a policy makers’ perspective, future programs should focus on not only non–financial strategies like educational opportunities or adequate equipment but also financial incentives. In contrast to several studies that determined that financial incentives were not the most powerful policy levers to attract medical students to rural locations [12, 25, 27, 30, 32, 35, 37, 46], our findings, along with other studies [9, 36, 47],suggesting that monthly income had a significant impact on job choices.

The WTP index was used as an estimate of the minimum compensation acceptable to healthcare students. The WTP results from the sub–group analysis for medical students revealed that women would pay more money for a harmonious patient–doctor relationship than men. As a result, female doctors could be absorbed into medical teams to improve patient–doctor relationships. Policymakers could target interventions to different sub–groups of students or at least consider their differential impact in their planning.

Our research using the WTP index also found that medical students from a rural background would pay more in exchange for all attributes of the hypothesized work, with the exception of rural job location. This finding is in line with previous research [25], but is somewhat at odds with a similar study in India [48]. It follows that the rural shortage of healthcare workers could be mitigated to some extent by a preferential admission of healthcare students from rural areas. On the other hand, we could infer that if students from a rural background can obtain the potential work, they may cherish the job opportunity. Thus, the preferential selection of rural students can be an effective strategy to attract more healthcare students to locate to rural areas. Furthermore, the findings lend support to the claim that student selection policies are important tools that can be used to achieve the stated policy objectives. For example, policy makers could devise a scheme whereby scholarships and student loans are provided to rural students who are willing to accept a job in a rural area upon completing their studies.

The results of policy simulation indicate that if these incentives are provided, medical and nursing students would take up jobs in rural areas. From a policy perspective, a higher salary would have the largest effect on recruitment, concurring with previous studies conducted in poorer areas [1, 35]. Consequently, policy makers should attach greater importance to the recruitment of nurses, who would be more sensitive to policy interventions although they were unwilling to locate to rural areas at the base line. Some scholars have argued that packages of interventions are essential for improving the distribution of human resources for health, and DCEs are one of the few methods available for comparing such packages [17]. This study examined the likely effect of incentive packages on the probability of attracting students to rural areas, and confirmed the importance of developing incentive packages to achieve the stated aims.

This study had several limitations. First, although the stated preference method used allowed us to determine relative strengths of the different attributes, it will not be able to fully anticipate the decisions that will eventually be made by participants in real life situations. In order to track their actual choices, we asked respondents to provide their telephone numbers on a voluntary basis. Second, the diverse intrinsic and extrinsic factors influencing job choices in real life change over time, which needs to be considered when designing recruitment policies. Third, this research was conducted in Guizhou province, so policy makers should deliberate whether the findings can be generalized to other places. Fourth, our research did not consider the costs associated with each type of incentives, which plays an important factor in any incentive strategy adopted by policy–makers. Thus, further research should focus on determining the expected cost of implementing the different policies to choose the most effective.

In conclusion, this study will help develop human resource priorities for health system reforms currently on the policy agenda in China. Our results suggest a variety of possibilities to improve doctors and nurses’ deployment in rural settings, such as increasing salaries, facilitating harmonious patient–doctor relationships, and eliminating the disparities between Bianzhi and contract–based work. Meanwhile, subgroup analyses indicate that one uniform recruitment policy is not recommended.

## Supplementary Information

**Additional file 1.**

**Additional file 2.**

## Data Availability

The datasets used and/or analyzed during the current study are available from the authors on reasonable request.
