# Supplementary material for: Job preferences of medical and nursing students seeking employment in rural China: a discrete choice experiment
Source: BMC Med Educ. 2021 Mar 5;21:146. doi: 10.1186/s12909-021-02573-3 (PMC7934374; doi:10.1186/s12909-021-02573-3)
Supplement: Supplementary file 1 — Additional file 1. [file 12909_2021_2573_MOESM1_ESM.docx]

**Supplementary files**

**Job preferences of medical and nursing students seeking employment in rural China: a discrete choice experiment**

Meiling Bao^1,2^ and Cunrui Huang^1,3*^

1 School of Public Health, Sun Yat-sen University, Guangzhou, China

2 School of Public Health, Guizhou Medical University, Guiyang, China

3 School of Public Health, Zhengzhou University, Zhengzhou, China

**^*^**Address correspondence to Cunrui Huang, School of Public Health, Sun Yat-sen University, Zhongshan Road #2, Guangzhou 510080, China. Email: [huangcr@mail.sysu.edu.cn](mailto:huangcr@mail.sysu.edu.cn).

**TableS1 Counties or districts interviewed in Guizhou Province, China**

| **County/District** | **GDP^a^**  **(RMB100 million)** | **Facility Numbers** | **Interviewee Numbers** |
| --- | --- | --- | --- |
| Zhijin | 154.41 | 2 | 10 |
| Hezhang | 112.06 | 2 | 9 |
| Weining | 189.37 | 1 | 2 |
| Zhongshan | 384.69 | 2 | 11 |
| Shuicheng | 206.29 | 2 | 9 |

GDP, gross domestic product.

The average GDP of 44 counties (districts) in Guizhou province was RMB196.32 (100 million) in 2015.

RMB￥1= USD $0.1479, in 2017.

^a^Data from Guizhou Statistical Yearbook 2015[1].

1. Guizhou Statistical Yearbook. [Available from: http//:<www.gz.stats.gov.cn/>. Accessed 20 July 2017.

**Figure S1 Example of a choice set**

| **Job** | **Education Opportunity** | **Transportation** | **Salary** | **Job Location** | **Workload** | **Essential Equipment** | **Patient–doctor Relationships** | **Bianzhi** |
| --- | --- | --- | --- | --- | --- | --- | --- | --- |
| **A** | Once every five years | Convenient | RMB3000yuan/month | City | 40 hours per week | Adequate | No(quarrel, physical conflict and suit) | Have |
| **B** | Once a year | Inconvenient | RMB7000yuan/month | Villages and towns | 60 hours per week | Inadequate | Quarrel | No |

Which job do you prefer: ____

**Table S2 Willingness to pay^a^ for medical students of sex and background difference**

| Variable | Female | Male | Rural | Urban |
| --- | --- | --- | --- | --- |
| Education opportunity |  |  |  |  |
| Once every two years | 9  (-426, 445)^b^ | 50  (-427, 529) | 236  (-52, 525) | 18  (-315, 278) |
| Once a year | 584  (110, 1060) | 615  (92, 1137) | 544  (228, 861) | 484  (154, 815) |
| Transportation |  |  |  |  |
| Convenient | 614  (277, 950) | 694  (322,1066) | 1112  (880, 1345) | 606  (364, 850) |
| Job location |  |  |  |  |
| City | 428  (-99, 956) | 657  (69, 1245) | 495  (138, 851) | 797  (415, 1179) |
| Workload |  |  |  |  |
| 40 hours per week | 367  (-69, 803) | 786  (293, 1278) | 600  (302, 900) | 557  (232, 883) |
| 60 hours per week | -449  (-899,-2) | -240  (-733, 253) | -564  (-862, -266) | -339  (-702, -96) |
| Essential equipment |  |  |  |  |
| Adequate | 1307  (958, 1657) | 782  (408, 1158) | 1184  (949, 1417) | 706  (468, 943) |
| Patient–doctor relationships |  |  |  |  |
| Quarrel | -1695  (-2224, -1166) | -885  (-1444, -326) | -1394  (-1738, -1050) | -956  (-1320, -592) |
| Physical conflict | -3946  (-4532, -3359) | -2396  (-2997, -1795) | -2741  (-3118, -2364) | -2193  (-2589, -1797) |
| Suit | -3681  (-4295, -3067) | -2268  (-2907, -1630) | -2818  (-3216, -2418) | -2038  (-2442, -1634) |
| Bianzhi |  |  |  |  |
| Have Bianzhi | 1304  (952, 1657) | 1419  (1030, 1809) | 1898  (1653, 2143) | 1001  (757, 1245) |

^a^RMB per month. RMB￥1= USD$ 0.1479, in 2017.

^b^95% confidence intervals in parentheses, the confidence intervals are calculated with the nlcom–command in Stata.
