# Supplementary material for: Job preferences of medical and nursing students seeking employment in rural China: a discrete choice experiment
Source: BMC Med Educ. 2021 Mar 5;21:146. doi: 10.1186/s12909-021-02573-3 (PMC7934374; doi:10.1186/s12909-021-02573-3)
Supplement: Supplementary file 2 — Additional file 2. [file 12909_2021_2573_MOESM2_ESM.docx]

Questionnaire file

**Job preferences of medical and nursing students seeking employment in rural China: a discrete choice experiment**

Meiling Bao^1,2^ and Cunrui Huang^1,3*^

1 School of Public Health, Sun Yat-sen University, Guangzhou, China

2 School of Public Health, Guizhou Medical University, Guiyang, China

3 School of Public Health, Zhengzhou University, Zhengzhou, China

**^*^**Address correspondence to Cunrui Huang, School of Public Health, Sun Yat-sen University, Zhongshan Road #2, Guangzhou 510080, China. Email: [huangcr@mail.sysu.edu.cn](mailto:huangcr@mail.sysu.edu.cn).

CODE：______

QUESTIONNAIRE ABOUT JOB PREFERENCES OF MEDICAL AND NURSING STUDENTS

Dear students:

The questionnaire survey would be implemented to medical and nursing students in medical school, which is about the job preferences between different simulated works and can be chosen to finish on the anonymous and voluntary basis.

**Basic personal information**

**Sex**：______

**Age**：______

**Background(Urban or Rural)**： ______

**Major**：______________________________

**Job preferences**

Imagine you will graduate from the college, and have been offered two jobs（Job A and Job B）.

In each of the twenty choices, you are asked to choose between two job positions. Each job differs with respect to a number of characteristics.

| **Job** | **Education Opportunity** | **Transportation** | **Salary** | **Job Location** | **Workload** | **Essential Equipment** | **Patient–doctor Relationships** | **Bianzhi** |
| --- | --- | --- | --- | --- | --- | --- | --- | --- |
| **A** | Once every five years | Convenient | RMB3000yuan/month | City | 40 hours per week | Adequate | No(quarrel, physical conflict and suit) | Have |
| **B** | Once a year | Inconvenient | RMB7000yuan/month | City | 60 hours per week | Inadequate | Quarrel | No |

**Choice 1** Which job do you prefer: ____

| **Job** | **Education Opportunity** | **Transportation** | **Salary** | **Job Location** | **Workload** | **Essential Equipment** | **Patient–doctor Relationships** | **Bianzhi** |
| --- | --- | --- | --- | --- | --- | --- | --- | --- |
| **A** | Once every five years | Convenient | RMB3000yuan/month | Villages and towns | 60 hours per week | Adequate | Quarrel | Have |
| **B** | Once every two years | Inconvenient | RMB5000yuan/month | Villages and towns | 40 hours per week | Inadequate | Physical conflict | Have |

**Choice 2** Which job do you prefer: ____

| **Job** | **Education Opportunity** | **Transportation** | **Salary** | **Job Location** | **Workload** | **Essential Equipment** | **Patient–doctor Relationships** | **Bianzhi** |
| --- | --- | --- | --- | --- | --- | --- | --- | --- |
| **A** | Once every two years | Convenient | RMB5000yuan/month | Villages and towns | 40 hours per week | Inadequate | No(quarrel, physical conflict and suit) | No |
| **B** | Once every five years | Convenient | RMB9000yuan/month | Villages and towns | 60 hours per week | Adequate | Physical conflict | Have |

**Choice 3** Which job do you prefer______

| **Job** | **Education Opportunity** | **Transportation** | **Salary** | **Job Location** | **Workload** | **Essential Equipment** | **Patient–doctor Relationships** | **Bianzhi** |
| --- | --- | --- | --- | --- | --- | --- | --- | --- |
| **A** | Once every two years | Inconvenient | RMB9000yuan/month | Villages and towns | 40 hours per week | Adequate | Physical conflict | No |
| **B** | Once a year | Convenient | RMB3000yuan/month | City | 50 hours per week | No(quarrel | No(quarrel, physical conflict and suit) | No |

**Choice 4** Which job do you prefer______

| **Job** | **Education Opportunity** | **Transportation** | **Salary** | **Job Location** | **Workload** | **Essential Equipment** | **Patient–doctor Relationships** | **Bianzhi** |
| --- | --- | --- | --- | --- | --- | --- | --- | --- |
| **A** | Once every two years | Convenient | RMB3000yuan/month | Villages and towns | 60 hours per week | Inadequate | Quarrel | Have |
| **B** | Once every five years | Inconvenient | RMB7000yuan/month | Villages and towns | 50 hours per week | Adequate | No(quarrel, physical conflict and suit) | No |

**Choice 5** Which job do you prefer______

| **Job** | **Education Opportunity** | **Transportation** | **Salary** | **Job Location** | **Workload** | **Essential Equipment** | **Patient–doctor Relationships** | **Bianzhi** |
| --- | --- | --- | --- | --- | --- | --- | --- | --- |
| **A** | Once a year | Convenient | RMB5000yuan/month | City | 40 hours per week | Adequate | Quarrel | Have |
| **B** | Once every two years | Inconvenient | RMB9000yuan/month | City | 50 hours per week | Inadequate | No(quarrel, physical conflict and suit) | No |

**Choice 6** Which job do you prefer______

| **Job** | **Education Opportunity** | **Transportation** | **Salary** | **Job Location** | **Workload** | **Essential Equipment** | **Patient–doctor Relationships** | **Bianzhi** |
| --- | --- | --- | --- | --- | --- | --- | --- | --- |
| **A** | Once every five years | Convenient | RMB7000yuan/month | City | 40 hours per week | Adequate | Physical conflict | Have |
| **B** | Once every two years | Inconvenient | RMB3000yuan/month | City | 50 hours per week | Inadequate | Suit | No |

**Choice 7** Which job do you prefer______

| **Job** | **Education Opportunity** | **Transportation** | **Salary** | **Job Location** | **Workload** | **Essential Equipment** | **Patient–doctor Relationships** | **Bianzhi** |
| --- | --- | --- | --- | --- | --- | --- | --- | --- |
| **A** | Once every two years | Convenient | RMB9000yuan/month | Villages and towns | 40 hours per week | Inadequate | Physical conflict | No |
| **B** | Once a year | Inconvenient | RMB7000yuan/month | City | 60 hours per week | Adequate | Quarrel | Have |

**Choice 8** Which job do you prefer______

| **Job** | **Education Opportunity** | **Transportation** | **Salary** | **Job Location** | **Workload** | **Essential Equipment** | **Patient–doctor Relationships** | **Bianzhi** |
| --- | --- | --- | --- | --- | --- | --- | --- | --- |
| **A** | Once a year | Convenient | RMB7000yuan/month | City | 40 hours per week | Adequate | Physical conflict | No |
| **B** | Once every five years | Inconvenient | RMB5000yuan/month | Villages and towns | 50 hours per week | Inadequate | Suit | Have |

| **Job** | **Education Opportunity** | **Transportation** | **Salary** | **Job Location** | **Workload** | **Essential Equipment** | **Patient–doctor Relationships** | **Bianzhi** |
| --- | --- | --- | --- | --- | --- | --- | --- | --- |
| **A** | Once every five years | Inconvenient | RMB7000yuan/month | City | 60 hours per week | Inadequate | No(quarrel, physical conflict and suit) | No |
| **B** | Once a year | Convenient | RMB5000yuan/month | Villages and towns | 50 hours per week | Adequate | Quarrel | Have |

**Choice 9** Which job do you prefer______

| **Job** | **Education Opportunity** | **Transportation** | **Salary** | **Job Location** | **Workload** | **Essential Equipment** | **Patient–doctor Relationships** | **Bianzhi** |
| --- | --- | --- | --- | --- | --- | --- | --- | --- |
| **A** | Once a year | Inconvenient | RMB5000yuan/month | City | 40 hours per week | Inadequate | Physical conflict | Have |
| **B** | Once every two years | Convenient | RMB3000yuan/month | Villages and towns | 50 hours per week | Adequate | No(quarrel, physical conflict and suit) | No |

**Choice 10** Which job do you prefer______

**Choice 11** Which job do you prefer______

| **Job** | **Education Opportunity** | **Transportation** | **Salary** | **Job Location** | **Workload** | **Essential Equipment** | **Patient–doctor Relationships** | **Bianzhi** |
| --- | --- | --- | --- | --- | --- | --- | --- | --- |
| **A** | Once a year | Inconvenient | RMB9000yuan/month | Villages and towns | 40 hours per week | Inadequate | No(quarrel, physical conflict and suit) | Have |
| **B** | Once every two years | Convenient | RMB7000yuan/month | City | 50 hours per week | Adequate | Physical conflict | No |

**Choice 12** Which job do you prefer______

| **Job** | **Education Opportunity** | **Transportation** | **Salary** | **Job Location** | **Workload** | **Essential Equipment** | **Patient–doctor Relationships** | **Bianzhi** |
| --- | --- | --- | --- | --- | --- | --- | --- | --- |
| **A** | Once every five years | Inconvenient | RMB7000yuan/month | Villages and towns | 50 hours per week | Inadequate | No(quarrel, physical conflict and suit) | No |
| **B** | Once a year | Convenient | RMB9000yuan/month | City | 40 hours per week | Adequate | Suit | Have |

**Choice 13** Which job do you prefer______

| **Job** | **Education Opportunity** | **Transportation** | **Salary** | **Job Location** | **Workload** | **Essential Equipment** | **Patient–doctor Relationships** | **Bianzhi** |
| --- | --- | --- | --- | --- | --- | --- | --- | --- |
| **A** | Once every two years | Convenient | RMB7000yuan/month | Villages and towns | 40 hours per week | Inadequate | Suit | No |
| **B** | Once a year | Inconvenient | RMB9000yuan/month | City | 60 hours per week | Adequate | No(quarrel, physical conflict and suit) | Have |

**Choice 14** Which job do you prefer______

| **Job** | **Education Opportunity** | **Transportation** | **Salary** | **Job Location** | **Workload** | **Essential Equipment** | **Patient–doctor Relationships** | **Bianzhi** |
| --- | --- | --- | --- | --- | --- | --- | --- | --- |
| **A** | Once every five years | Inconvenient | RMB3000yuan/month | City | 60 hours per week | Inadequate | Physical conflict | No |
| **B** | Once a year | Convenient | RMB9000yuan/month | City | 40 hours per week | Adequate | Suit | Have |

**Choice15** Which job do you prefer______

| **Job** | **Education Opportunity** | **Transportation** | **Salary** | **Job Location** | **Workload** | **Essential Equipment** | **Patient–doctor Relationships** | **Bianzhi** |
| --- | --- | --- | --- | --- | --- | --- | --- | --- |
| **A** | Once a year | Inconvenient | RMB3000yuan/month | Villages and towns | 50 hours per week | Adequate | Physical conflict | No |
| **B** | Once every two years | Convenient | RMB7000yuan/month | Villages and towns | 60 hours per week | Inadequate | Suit | Have |

**Choice 16** Which job do you prefer______

| **Job** | **Education Opportunity** | **Transportation** | **Salary** | **Job Location** | **Workload** | **Essential Equipment** | **Patient–doctor Relationships** | **Bianzhi** |
| --- | --- | --- | --- | --- | --- | --- | --- | --- |
| **A** | Once every five years | Convenient | RMB7000yuan/month | Villages and towns | 50 hours per week | Inadequate | Suit | Have |
| **B** | Once every two years | Inconvenient | RMB3000yuan/month | Villages and towns | 40 hours per week | Adequate | Quarrel | No |

**Choice 17** Which job do you prefer______

| **Job** | **Education Opportunity** | **Transportation** | **Salary** | **Job Location** | **Workload** | **Essential Equipment** | **Patient–doctor Relationships** | **Bianzhi** |
| --- | --- | --- | --- | --- | --- | --- | --- | --- |
| **A** | Once every five years | Inconvenient | RMB5000yuan/month | City | 60 hours per week | Adequate | Physical conflict | Have |
| **B** | Once a year | Convenient | RMB9000yuan/month | Villages and towns | 50 hours per week | Inadequate | Quarrel | No |

**Choice 18** Which job do you prefer______

| **Job** | **Education Opportunity** | **Transportation** | **Salary** | **Job Location** | **Workload** | **Essential Equipment** | **Patient–doctor Relationships** | **Bianzhi** |
| --- | --- | --- | --- | --- | --- | --- | --- | --- |
| **A** | Once every two years | Convenient | RMB9000yuan/month | City | 50 hours per week | Adequate | Suit | No |
| **B** | Once a year | Inconvenient | RMB7000yuan/month | Villages and towns | 40 hours per week | Inadequate | Quarrel | Have |

**Choice 19** Which job do you prefer______

| **Job** | **Education Opportunity** | **Transportation** | **Salary** | **Job Location** | **Workload** | **Essential Equipment** | **Patient–doctor Relationships** | **Bianzhi** |
| --- | --- | --- | --- | --- | --- | --- | --- | --- |
| **A** | Once every five years | Convenient | RMB3000yuan/month | City | 60 hours per week | Inadequate | Quarrel | No |
| **B** | Once every two years | Inconvenient | RMB9000yuan/month | City | 50 hours per week | Adequate | No(quarrel, physical conflict and suit) | Have |

**Choice 20** Which job do you prefer______

The end!

Thank you for your participation very much!

Your telephone number(Optional)：__________________

date：______
